# Meiotic drive adaptive testes enlargement during early development in the stalk-eyed fly

**DOI:** 10.1098/rsbl.2022.0352

**Published:** 2022-11-30

**Authors:** Sasha L. Bradshaw, Lara Meade, Jessica Tarlton-Weatherall, Andrew Pomiankowski

**Affiliations:** ^1^ Department of Genetics, Evolution and Environment, University College London, Gower Street, London WC1E 6BT, UK; ^2^ CoMPLEX, University College London, Gower Street, London WC1E 6BT, UK

**Keywords:** accessory gland, meiotic drive, sex ratio distortion, sexual selection, stalk-eyed fly, testis

## Abstract

The sex ratio (SR) X-linked meiotic drive system in stalk-eyed flies destroys Y-bearing sperm. Unlike other SR systems, drive males do not suffer fertility loss. They have greatly enlarged testes which compensate for gamete killing. We predicted that enlarged testes arise from extended development with resources re-allocated from the accessory glands, as these tend to be smaller in drive males. To test this, we tracked the growth of the testes and accessory glands of wild-type and drive males over 5–6 weeks post-eclosion before males attained sexual maturity. Neither of the original predictions is supported by these data. Instead, we found that the drive male testes were enlarged at eclosion, reflecting a greater allocation of resources to the testes during pupation. Testes grow at a higher rate during early adult development in drive males, but there was no evidence that this retards the growth of the accessory glands. Further experiments are proposed to investigate whether smaller accessory glands only arise in drive males post-copulation or when flies are subjected to nutritional stress. Our experimental findings support the idea that enlarged testes in drive males arise as an adaptive allocation of resources to traits that enhance male reproductive success.

## 1. Introduction

Mendel's first law of equal segregation holds for most nuclear genes. This fair segregation can be subverted by meiotic drivers that gain a transmission advantage, often in conflict with the rest of the genome [1–3]. Male meiotic drivers are genetically and mechanistically diverse, but all result in the death or disabling of non-carrier sperm [4]. Many examples exist, both of autosomal (e.g. SD in *Drosophila melanogaster* [5,6] and *t* locus in mice [7,8]) and sex-linked origin (e.g. sex ratio (SR) in *Drosophila simulans* [9,10] and *Slx*/*Sly* in mice [11]). Owing to the dysfunction of wild-type sperm, meiotic drive detrimentally impacts male fertility [12,13], with drive sperm typically being less effective under sperm competition [8,9,13]. In addition, meiotic drive is often associated with viability reduction in both males and females [14]. Sex chromosome drive is also associated with various costs through the distortion of the population sex ratio [15–18], which can potentially lead to local extinction [19–21].

In response to these costs, host nuclear genes have been selected to resist drive or counter its deleterious effects [22–24]. A common response is the evolution of drive suppressors [24–26]. A number of putative behavioural adaptations are known. In the *t*-haplotype system in mice, juvenile dispersal is enhanced in *t* heterozygotes which reduces the probability of lethal homozygosity [27]. Another example is the theoretical prediction that females should mate polyandrously to decrease the success of drive-bearing sperm [28]. In alignment with this, experimental populations of *Drosophila pseudoobscura* exposed to a high frequency of SR meiotic drive evolved increased female remating [29], although there is no evidence that variation in drive frequency is a major factor determining female mating rate in wild populations [29]. It has also been suggested that mate choice might allow females to discriminate against drive-carrying males, either through the pleiotropic effects of drive or via signals of genetic quality where drive is associated with reduced viability [14,30]. There are some tentative examples, such as the major histocompatibility complex linked with the *t* haplotype in mice [31], and reduced eyespan in male stalk-eyed flies where female preference favours longer eyespan [32]. However again, there is no evidence that the presence of drive has led to the strengthening of mate preferences.

We investigated evidence for an adaptive response in reproductive organ size to X-linked drive (SR) in the Malaysian stalk-eyed fly, *Teleopsis dalmanni*. Previous work has shown that SR males deliver the same number of sperm per ejaculate [33] and do not suffer fertility loss compared to wild-type males [34]. This reflects a massive increase in the size of their testes, which are approximately 26% larger than wild-type [34]. This could be owing to a resource trade-off with the accessory glands, which are reduced in SR males [34]. In order to test this hypothesis, the testes and accessory glands of SR and wild-type males were dissected and measured over a series of developmental timepoints from eclosion to beyond the point of sexual maturity [35–37] to determine interactions in the growth profiles of these reproductive organs.

## 2. Material and methods

A wild-type, standard (ST) stock was collected in 2005 from the Ulu Gombak Valley, Peninsular Malaysia (by A. Pomiankowski and S. Cotton). Flies with the X^SR^ genotype were collected in 2012 from the same location and since 2019 have been maintained as a homozygous SR stock [16,38]. Experimental ST males (X^ST^/Y) were collected on egglays (Petri dish with damp cotton and sweetcorn) from cages housing X^ST^/X^ST^ females and X^ST^/Y males. The egglays were incubated at 25°C and the emerging flies were collected daily. Males were housed by emergence date and females were discarded. The same procedure was followed to collect SR males (X^SR^/Y) from cages housing homozygous X^SR^/X^SR^ females and X^ST^/Y males.

Two experiments were conducted following the same method. The first experiment performed dissections over a longer period from day 0 (eclosion) to day 56 (long dataset). Flies were dissected on days 0, 1, 4, 8, 12, 16, 20, 34 and 56 (*n* = 24–53 per time point; electronic supplementary material, tables S1 and S2). A follow-up experiment was carried out with more intense measurements from day 11 to day 25 (short dataset), with dissections on days 11, 13, 15, 17, 19, 21, 23 and 25 (*n* = 37–53 per time point; electronic supplementary material, tables S3 and S4). The thorax and eyespan of ice-anaesthetized flies were measured prior to dissection, using an Infinity Capture video microscope attached to a computer equipped with NIH image software (FIJI (ImageJ), version 2.1.0/1.53c). The thorax was measured from the prothorax anterior tip, along the midline ending at the joint in-between the thorax and metathoracic legs [36]. Eyespan was measured from the outer tips of the eyes adjacent to where the stalk joins the eye bulb [38]. Flies were then dissected in 15 µl of phosphate-buffered saline (PBS) using 5 mm forceps on a glass slide under the stereomicroscope. The testes and accessory glands were isolated then untangled and uncoiled without causing rupture or damage. Excess material such as external cuticle was removed from the slide to prevent distortion of the image. Another 15 µl of PBS was added before adding a glass cover. Images were taken using a differential interference contrast microscope on QCapture Pro imaging software at either x5 or x10 magnification. The polygon selection tool in ImageJ was used to take area measurements for both the testes and accessory glands, by tracing around the outline of the organs.

### (a) Statistical analysis

All statistical analyses were performed in R (v.1.4.1103). Linear regression models were used to identify differences in reproductive organ size between genotypes. Models included genotype, age (days), thorax size (body size) and relative eyespan. A stepwise build-up was used to add terms that improved the model fit. Terms that did not improve the model fit were discarded. The morphological traits of thorax size and relative eyespan were added as covariates as they are known to differ between genotypes, and correlate with reproductive organ size in mature adult flies [34]. Relative eyespan was calculated from the residuals using a linear regression model after taking into account thorax size, as these traits are strongly collinear [39]. To determine trade-offs between the development of the testes and accessory glands with other morphological traits, interaction terms were tested. Specifically, break-point analysis was conducted to investigate the interaction between genotype and age (days) on the testes as a proxy of growth rate. Mean and standard error trait sizes (mm) are reported throughout. See the electronic supplementary material for all models.

## 3. Results

### (a) Body size and eyespan

In these holometabolous insects, body size (thorax) and eyespan are fixed at eclosion. In the long dataset, the body size of SR (mean ± s.e. = 2.324 ± 0.012) and ST males (2.352 ± 0.013) did not differ (*F*_1,367_ = 2.651, *p* = 0.104; electronic supplementary material, model 1). The eyespan of SR (7.872 ± 0.056) was smaller than ST males (8.095 ± 0.061, *F*_1,367_ = 7.266, *p* < 0.001; electronic supplementary material, model 3), and this held after controlling for body size (*F*_1,366_ = 5.253, *p* < 0.010; electronic supplementary material, model 4). In the short dataset, the body size of SR (2.441 ± 0.009) was smaller than ST males (2.511 ± 0.009; *F*_1,355_ = 29.327, *p* < 0.001; electronic supplementary material, model 5). Once more, eyespan was smaller in SR (8.382 ± 0.034) than ST males (8.753 ± 0.032; *F*_1,355_ = 61.941, *p* < 0.001; electronic supplementary material, model 7), and this held after controlling for body size (*F*_1,354_ = 31.197, *p* < 0.001; electronic supplementary material, model 8).

### (b) Testes

**Figure 1.**
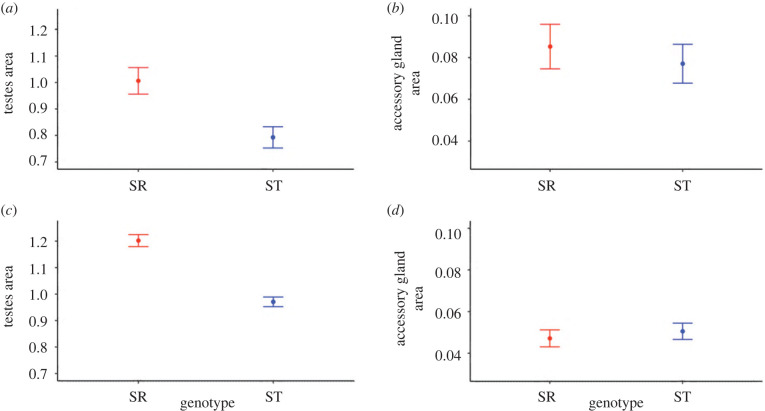
Mean ± s.e. testes area (mm^2^) for SR (red) and ST (blue) in the (*a*) long (0–56 days) and (*c*) short datasets (11–25 days), and similar measures for accessory gland area in the (*b*) long and (*d*) short datasets.

**Figure 2.**
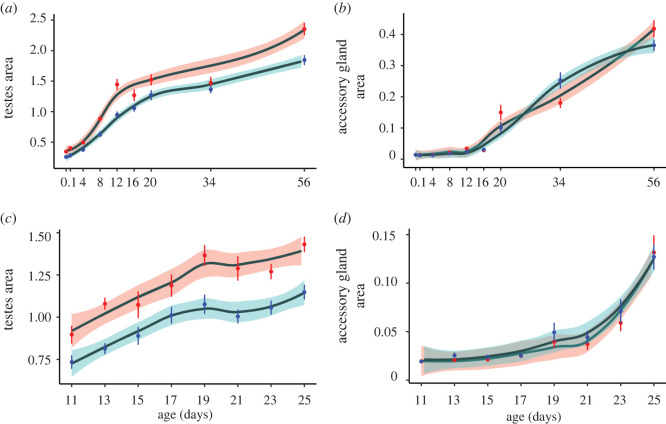
Growth curves across age (days since eclosion) for SR (red) and ST (blue) testes area (mm^2^, shaded area represents ± s.e.) in the (*a*) long and (*c*) short datasets, and for accessory gland area in the (*b*) long and (*d*) short datasets. Growth curves were fitted using the ‘loess’ function of the ‘ggplot2’ package in R (v1.4.1103).

Given these findings, thorax and relative eyespan were used as covariates in the following analyses. Controlling for the day of dissection, SR had larger testes (1.006 ± 0.050) than ST males (0.793 ± 0.040; *F*_1,355_ = 49.626, *p* < 0.001; figure 1*a*; electronic supplementary material, model 12), and this held after controlling for body size and relative eyespan (*F*_1,353_ = 57.275, *p* < 0.001; electronic supplementary material, model 13) in the long dataset. The same was the case over the restricted timeframe of the short dataset (SR: 1.202 ± 0.023, ST: 0.97 ± 0.018; *F*_1,344_ = 84.038, *p* < 0.001; figure 1*c*; electronic supplementary material, model 15), and again after controlling for body size and relative eyespan (*F*_1,342_ = 107.194, *p* < 0.001; electronic supplementary material, model 16). Considering individual time points separately, SR male testes were larger on days 0, 1, 4, 8, 12, 20 and 56 (*p* < 0.05) but not on days 16 and 34 (*p* > 0.05; figure 2*a*; electronic supplementary material, table S1) in the long dataset. When repeated at the higher sample size in the short dataset, SR male testes were larger on days 11, 13, 17, 19, 21, 23 and 25 (*p* < 0.05) but marginally not on day 15 (*p* = 0.052; figure 2*c*; electronic supplementary material, table S3).

In addition, there was an interaction between the day of dissection (age) and genotype in the long dataset (*F*_1, 353_ = 8.135, *p* < 0.010; electronic supplementary material, model 17), with a stronger rate of increase in testes area among SR males (figure 2*a*). The difference in growth rate was in early (days 0–20) but not in later development (days 12–56; electronic supplementary material, table S5). This was confirmed using break-point analysis, which identified a higher early growth rate that completed earlier in SR males (SR slope = 0.082, break-point = 12 days; ST slope = 0.054, break-point = 18 days), followed by a period in which the growth rates were more similar (SR slope = 0.023, ST slope = 0.016). As the short dataset covered the middle range of development (days 11–25), it showed no difference in the rate of testes growth between SR and ST males (*F*_1, 342_ = 1.108, *p* = 0.293; electronic supplementary material, model 18).

### (c) Accessory glands

Accessory gland growth contrasted with the testes as there was no overall difference between SR and ST males in either the long (SR: 0.085 ± 0.012, ST: 0.077 ± 0.009, *F*_1,315_ = 0.339, *p* = 0.561; figure 1*b*; electronic supplementary material, model 19) or short dataset (SR: 0.047 ± 0.004, ST 0.051 ± 0.004, *F*_1,345_ = 0.358, *p* = 0.550; figure 1*d*; electronic supplementary material, model 22), or after controlling for body size and relative eyespan (long: *F*_1,312_ = 0.929, *p* = 0.336; electronic supplementary material, model 21; short: *F*_1,342_ = 0.132, *p* = 0.716; electronic supplementary material, model 24). Likewise, there was no difference on individual dissection days in either dataset (*p* > 0.05; figure 2*b,d*; electronic supplementary material, tables S2 and S4), nor was there an interaction between day of dissection (age) and genotype in either dataset (long: *F*_1, 312_ = 1.677, *p* = 0.196; electronic supplementary material, model 25; short: *F*_1, 342_ = 1.108, *p* = 0.293; electronic supplementary material, model 26).

## 4. Discussion

Male carriers of SR meiotic drive in *T. dalmanni* experience reduced sperm production owing to the dysfunction of non-carrier sperm [33]. They compensate for this with enlarged testes. The experiments here show that testes area is larger at eclosion (day 0), 33.72% larger than wild-type males, which reflects greater resource allocation during SR male pupal development. In addition to eclosion size being larger, SR male testes show higher growth rates during the early period of adult development up to approximately day 20, beyond which growth rates are similar to those in wild-type males (figure 2). These findings suggest that the pre-eclosion and early development enlargement of the testes is an adaptation to compensate for the future loss of sperm caused by meiotic drive, allowing adult male sperm production and fertility to be maintained at the same level as wild-type males [34]. This is likely to be encoded on the X^SR^ chromosome that contains a set of inversions spanning most of its length [40], which would allow tight linkage to be maintained with the genes responsible for controlling drive.

These findings allow us to reject the hypothesis that greater SR testes area is a passive response to lower sperm production, as sperm bundles mature much later in development from 12 days post-eclosion [37]. Nor do they support the idea that the adult development of the testes occurs for a longer period in SR males. Both the individual day comparisons and break-point analyses indicate that the higher early growth rate of the SR testes is curtailed at the same time as, or even earlier than, ST males (figure 2*a*). Another possibility is that drive larvae and adults accumulate resources at a higher rate. This hypothesis seems unlikely, as all flies were provided with ad libitum food during larval and adult development and there is no obvious explanation as to why wild-type flies would feed at a lower rate. Currently, we have no direct evidence with which to test this idea, highlighting the necessity to assess it in further experiments.

More plausible is our previous hypothesis that the enlargement of the testes induced a resource trade-off with the accessory glands, which are smaller in SR adult males [34]. However, there was no evidence of a pupal resource allocation trade-off as the accessory glands were the same size in drive and wild-type males at eclosion. Nor was there evidence post-eclosion, as the greater growth rate of the testes in SR males did not depress accessory gland area in either dataset, despite the second ‘short dataset’ (days 11–25) being designed to hone-in on the period when both reproductive organs undergo rapid development. In fact, there was no explicit difference in accessory gland area at any development stage from eclosion to day 56. This finding contrasts with previous experimental work [34] and data from the field (A. Pomiankowski 2022, unpublished data) showing reduced accessory gland area in SR males. A possible explanation is that flies used in the present study were virgins. In the study by Meade *et al*. [34], dissections were performed on males, the majority of which had already mated with one or several females. Mating causes a decrease in accessory gland area [35,37], and we hypothesize that mated SR drive males may have a reduced capacity to replenish their accessory gland resources. There is indirect support for this idea, as SR males mate comparatively less than ST males when housed with multiple females as they take longer to re-mate [34].

An additional possibility concerns nutritional stress in adult development, which is known to reduce the accessory glands [35]. If the effect of adult nutritional stress is exacerbated in drive males, it might contribute to more severe reductions in resource availability for accessory gland growth. This could explain the finding of smaller accessory glands in drive males caught in the field, where food restriction is likely to be common. These hypotheses, on the effects of mating and nutritional stress, will be addressed in follow-up experiments.

## Data Availability

Data and data description are available from the Dryad Digital Repository: https://doi.org/10.5061/dryad.bk3j9kdfz [41]. The electronic supplementary material contains two sample *t*-tests comparing accessory gland area and testes area across genotypes (tables S1–S4), the age : growth rate interaction (table S5) and detailed statistical models [42].
